# Development, Implementation and First Evaluation of an Online Portal to Promote the Mental Health of University Students (me@JGU)

**DOI:** 10.3390/ijerph18031179

**Published:** 2021-01-28

**Authors:** Caroline Lutz-Kopp, Ursula Luka-Krausgrill, Bettina Honsbrok, Bozana Meinhardt-Injac, Maria Gropalis

**Affiliations:** 1 Mental Health Services for Students, Johannes Gutenberg University, 55122 Mainz, Germany; luka-krausgrill@t-online.de (U.L.-K.); bettina.honsbrok@uni-mainz.de (B.H.); maria.gropalis@uni-mainz.de (M.G.); 2 Department of Psychology, Catholic University of Applied Sciences Berlin, 10318 Berlin, Germany; bozana.meinhardt-injac@khsb-berlin.de

**Keywords:** mental health in university students, online unguided self-help, prevention, resilience, online portal

## Abstract

Background: University students encounter various stressors such as exam preparation, workload and economic concerns. Having to deal with a multitude of stressors can lead to mental health problems and have a negative impact on academic outcomes in students attending university. This paper describes the development and usability evaluation of an open and easily accessible online portal (me@JGU) designed to help students build skills they need to cope with common stressors and manage their own mental health. Methods: We developed a website that addresses the most common stressors among university students and offers strategies for dealing with difficult situations. Initial evaluation results were collected using website statistics and a short anonymous survey regarding the attractiveness and usability of the website. Results: Over an eight-month period, there were 5739 visitors, a total of 16,495 page views and 3748 downloads. The survey results indicate that me@JGU covers relevant topics and that the students like the layout. Conclusions: Online interventions promoting mental health of university populations are easily accessible and cost effective for large populations. They may prevent study difficulties, inform students about mental health and offer possible solutions. In addition, at-risk students can receive information about other relevant resources, and feel encouraged to access support and treatment.

## 1. Introduction

The time spent at university is an important developmental phase and holds many challenges for the students transitioning from adolescence to young adulthood [1]. For some years now, increasing attention has been drawn to the psychological stress on students. Studies using quantitative measures such as self-report inventories consistently report exams, assignments and workload, financial pressures as well as transitioning to university as typical stressors experienced by students (for an overview see [2]). A review on qualitative research concerning college student stressors found eight major categories: relationships (e.g., family, peer, faculty), lack of resources (e.g., time, money, support), living up to expectations, academic themes (e.g., coursework, exams), environment (e.g., unfamiliar), diversity (e.g., ethnicity, disability) and transitioning to the university setting [3].

A nationwide online survey at German universities with 18,214 participants identified exams, assignments, workload and meeting one’s own expectations as most common stressors among higher education students [4]. Additionally, struggling to organize university life and to have a good “study-life balance” were referred to as important stressors.

According to a study by Gusy and colleagues [5], the demands of studying in Germany include a high workload, poorly designed study programs and environments, as well as weak relationships with other students. Resources in studies, on the other hand, include social support and feedback on work results. In a university-wide online survey at the Johannes Gutenberg University Mainz (JGU) with more than 2400 participants, 24% of the students reported a high level of exhaustion, 55% had a remarkably low level of well-being and 58% reported a low level of stress resistance [6].

Chronic stress can be a trigger for mental disorders [7,8,9,10]. In the context of the WHO World Mental Health International College Student Initiative (WMH-ICS), which aims at developing an infrastructure to examine and improve the mental health of college students around the world [11], a recent cross-national study including 20,842 students from 24 universities in nine countries found a significant dose–response association between the extent of perceived stress and increased probability of having a mental disorder [12]. Another study of the WMH-ICS using self-report measures found that 31% of students fulfilled the criteria of being afflicted by a mental disorder in the last 12 months [13]. In Germany, according to health insurance company data, about 17–22% of students suffer from a mental disorder [14,15]. Matching these results, the number of clients at the Mental Health Services for Students at JGU has been rising continuously over the last years despite decreasing student numbers (2007: 548 to 2019: 910; [16], total number of students: approximately 31,300). 

Having a mental disorder can lead to a reduced ability to perform and study, which could result in interruptions, delays and even discontinuation of studies [17,18,19,20]. College students with mental disorders are twice as likely as other students to drop out without graduating [21,22,23]. Additionally, a substantial proportion of the students with mental disorders who continue their studies experience a negative impact on academic performance due to their mental problems [24,25,26,27].

In order to help students to complete their studies successfully, the promotion of their mental health is of particular importance to the universities’ counseling centers. Using internet-based interventions to treat various mental disorders (e.g., [28,29,30]) as well as to promote mental health for different psychological problem areas has been successfully implemented for the general population [31].

Programs directed at reducing students’ distress and promoting mental health typically include psychoeducation and exercises on the topics of activity and mood, motivation, dealing with thoughts and feelings, social relationships, stress management, perfectionism and self-esteem (for an overview, see [32]). Various randomized-controlled trials have shown them to be effective in reducing distress in college students [32,33,34,35,36,37]. Two recent systematic reviews and meta-analyses have yielded small to moderate intervention effects [32,33]: The study by Amanvermez and colleagues (2020) found a significant moderate effect size for stress management interventions for students with high-stress levels (*g* = 0.54; 95% CI (0.31, 0.78); *p* < 0.001), a small to-moderate effect size for depression (*g* = 0.46; 95% CI (0.16, 0.77); *p* = 0.003), a moderate-to-large effect for stress (*g* = 0.61; 95% CI (0.30, 0.93); *p* < 0.001), and a moderate effect for anxiety (*g* = 0.52; 95% CI (0.25, 0.78); *p* < 0.001; [33]). Harrer et al. [32] evaluated internet interventions for mental health in college students, and report small effects on depression (*g* = 0.18, 95% confidence interval (CI (0.08, 0.27)), anxiety (*g* = 0.27, 95% CI (0.13, 0.40)), and stress (*g* = 0.20, 95% CI (0.02, 0.38)). 

Another important advantage of web-based interventions is that they are easily accessible, and cost effective for large populations. 

However, the transferability of the results to naturalistic settings, such as routine care programs, has not yet been studied. 

The aim of this study was to develop an open and easily accessible online portal (me@JGU) to promote student’s mental health by helping them deal with common college students’ stressors. This website is meant specially to promote the students’ resources and self-help skills by teaching strategies for dealing with difficult (study-relevant) situations and critical life events. The strategies are all based on cognitive-behavioral therapy.

This article presents the development and implementation of the online portal (me@JGU) as well as the first evaluation results.

## 2. Materials and Methods

### 2.1. Background and Scope of the Project

The Mental Health Services for Students at JGU are open to all university’s students and are free of charge. We provide professional psychotherapeutic help in dealing with specific problems that may occur during studies (e.g., depressive symptoms, writing difficulties, test anxiety, stress). We applied for and realized me@JGU as a subproject within the university-wide LOB (“Lehren-Organisieren-Beraten”) project at JGU. The LOB project was funded by the Federal Ministry of Education and Research (Bundesministerium für Bildung und Forschung) and ran from 2017 until the end of 2020. It developed measures in three fields of action—teaching, organizing, advising—that focus on a sustainable improvement of study conditions. The aim was to support students in completing a successful course of study in terms of competence acquisition, personal development and the achievement of formal qualifications. It was a university-wide networked project with 30 individual projects in 9 departments and 10 central institutions.

### 2.2. Development: Website Creation

We named the website “me@JGU—mental fit durchs Studium”, which roughly translates to “me@JGU—staying mentally healthy during your studies”. (The “me” in “me@JGU” is a wordplay: on the one hand it represents an abbreviation of the German phrase “mental fit durchs Studium” (Engl. “mentally healthy during your studies”) and also is meant to stand for “I at JGU”.) At the beginning, we conducted an extensive literature research to identify typical stressors among university students and online interventions for mental health in student populations (for an overview, see [32]).

Based on our findings from the literature research, we then carried out a needs analysis to ensure that the content of the planned online portal appropriately addressed the needs of the students at JGU. We first assessed the current mental health of the students [6]. Second, we asked about typical stressful events in everyday university life and the students’ wishes regarding the content and the presentation of an online portal promoting mental health (unsubmitted data). The needs analysis has shown similar results as our literature research. Taking both sources of information together, we focused on the most common student stressors: (1) academic themes (i.e., exams, assignments, workload, organizing studies), (2) relationships, (3) transitioning to university, and (4) living up to one’s own expectations [2,3,4,5]. We did not include the stressors “financial resources”, “environment”, and “diversity” in our program, because there are other internal university offices at JGU that are specialized in these topics (e.g., coordination office for diversity or student union in case of financial needs). However, we linked theses offers on our website.

In the next step, the contents and the website were developed. The chapters were then gradually published on the website, evaluated, and revised. At the end of the development process, the online portal consisted of eight chapters. Each chapter is dedicated to one topic (see Table 1 for further information). Considering academic themes being the most often reported stressors for college students, three of our chapters aimed directly at reducing stress in this area in order to promote the students’ mental well-being: (a) the chapter “mastering exam stress” provides psychoeducative information and skills to improve exam preparation and learning strategies; (b) the chapter “well organized” contains information on implementing time schedules throughout the semester and how to balance studies with other important parts of life; (c) the chapter “defeating procrastination” provides strategies on how to stay motivated during studies.

In order to ease the transition to university and to improve the students’ relationships, the chapter “your social network” was compiled.

The chapters “strengthening self-esteem” and “finding goals” were developed in order to help students living up to their own expectations, and included information and strategies to reflect on their strengths and values. An additional advantage of the chapter “strengthening self-esteem” is that increasing an individual’s self-esteem might also increase their ability to cope with stress [38].

The two remaining chapters are meant to strengthen the students’ resilience to stress in a more general way [34]: (a) “promoting relaxation” targets with behavioral interventions the management of the physical stress reaction (e.g., tension); (b) “managing negative feelings and thoughts” aims to assist individuals in identifying and modifying dysfunctional beliefs that influence response to stimuli and subsequent physiological and psychological distress as well as successfully regulating emotions.

In the beginning, we planned to design the website ourselves and shoot the videos on our own. Due to the already implemented online counseling programs at the Mental Health Services for Students, the counseling team has the necessary technical skills. However, the website would then have looked very similar to the university’s teaching offerings. We were concerned that it would remind the students of coursework and therefore be less interesting and fun to work with. Furthermore, in order to address all students, the website was planned to function without our counseling support, which is known to lead to lower user numbers [29,39,40,41]. We decided to obtain professional support to ensure that the website would be visually attractive and stand out from other offers of the university to appeal to the students and keep them motivated. Nevertheless, the layout shows clearly that it is an offer of the university for its students (i.e., we used the university’s logo and added specific information concerning JGU and the responsibility for the content) in order to ensure the trustworthiness of the program. The layout and programming of the website was done by a design agency, in close cooperation with us (see Appendix A). The videos were produced with the support of the JGU’s Center for Audiovisual Production. Currently, the website can only be accessed by students and staff of JGU. Opening up the website for other universities is planned for the future.

**Figure 1:**
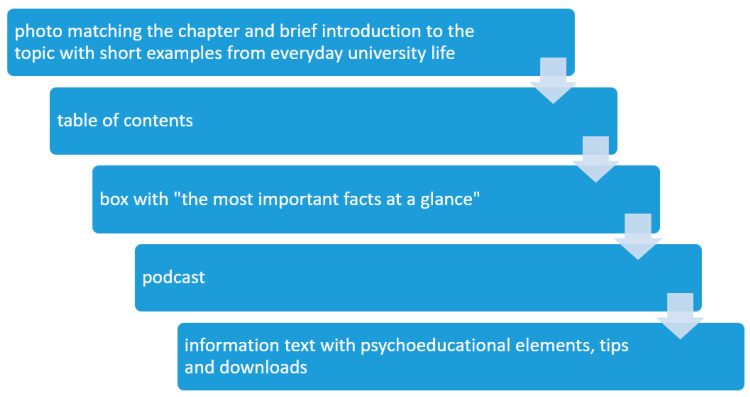
Structure of each chapter of the website (me@JGU).

At the time of data analysis, we had already published six of the planned eight chapters. (The publication of the last two chapters (“your social network”, “managing thoughts and feelings”) was unfortunately delayed due to the COVID-19 pandemic until the end of 2020. Thus, we are not able to report data on these two chapters.) In every chapter, we teach central competences that help to improve coping skills in stressful situations and events and, therefore, should strengthen students’ mental health. We offer information, tips and exercises on various topics in the form of texts, videos and audios. For a more in-depth confrontation with the topic, students have the possibility to download worksheets with exercises, tips and checklists as PDF files. Each chapter is structured in the same way: first there is a photo, followed by a brief introduction to the topic with short examples from everyday university life. Then, a table of contents is displayed, followed by a box with “the most important facts at a glance”. After that, the chapter is offered as a podcast, for those who prefer to listen to the content. Subsequently, the main information text with psychoeducational elements, tips and downloads follows (see Figure 1).

In order to establish a local connection to Mainz and JGU, we tried to relate all of the content and tips to the city and the university. In the chapter “promoting relaxation”, for example, there is a relaxation exercise in the form of a video that takes students on a walk through the JGU botanical garden. We also linked offers from other internal university offices at JGU (such as Career Service, student advisory services, university sport offers, advice in case of financial needs) in the corresponding chapters of the website. Furthermore, we recommended self-help literature.

### 2.3. Implementing the Website

On 24 October 2019, the Mental Health Services for Students of JGU participated in the World Mental Health Day with a day of action on campus to raise awareness of mental health issues students face. According to the motto “every 5th student is affected—keep an eye on your mental health”, the team of the counseling center informed students with a quiz about interesting facts concerning mental disorders. Within this action day, we also launched and promoted me@JGU. 

Further advertising measures were an information mail to all students (approximately 31,300 enrolled students in winter semester 2019/2020), an entry on JGU’s Facebook and Instagram channel, and various university departments reported on their websites about the launch of me@JGU. We also advertised on our own website as well as informed clients in individual counseling and workshops at the Mental Health Services for Students. In addition, we had posters and bookmarks with short tips such as “do it today” or “set priorities” made, which were given to students and sent to study offices and student advisors. Furthermore, a short video teaser introducing the website was shot and shown on different occasions. Additionally, an interview about the online portal was published in a local student newspaper (STUZ).

### 2.4. (First) Evaluation of the Concept

Although the ultimate aim in evaluating a new online mental health intervention may be to conduct randomized controlled trials (RCT), there are several distinct phases of evaluation that need to be considered before this stage [42]. Early stage evaluations usually focus on usability, and subsequent testing includes issues such as engagement and efficacy analyses. Perceived usefulness and perceived ease of use have proven to be fundamental determinants of user acceptance of information technology in general (for further information concerning the Technology Acceptance Model, see [43,44]), but also in understanding university students’ behavioral intention to use e-learning [45]. We therefore focused in our first evaluation on questions concerning this matter. The results reported here will influence further website adaptations. 

In order to investigate the appeal and use of the website, we collected various data anonymously without any information on the profile of the users (e.g., demographic data). The decision to collect only anonymous data in this early phase of the study was made in order to adhere to privacy protection guidelines according to the European General Data Protection Regulation. Next steps will involve evaluating the efficacy of the program in promoting mental health in more detail. 

#### 2.4.1. Website Usage

Collecting website usage data in order to evaluate web-based interventions provides information on levels of intervention use as well as typical navigation patterns through a website and represents an important addition to self-reporting [46]. It represents the most commonly used objective measure of engagement in the behavioral science literature [47]. The usually collected data is the number of log-ins, the number of completed intervention components, activity, and the time spent on the website. As our website is designed as an unguided self-help offer, we focused on evaluating the click numbers (i.e., numbers of log-ins), the time spent on the website, and downloads from the website via Matomo (https://matomo.org/) [48]. Matomo is an open source software for statistical analysis of user interaction. Information collected via Matomo is client (browser) based, stored on a server of JGU, and is compliant with the European General Data Protection Regulation.

#### 2.4.2. Short Survey

A short online survey regarding the attractiveness and the comprehensibility of the website was linked at the end of each chapter. 

The online questionnaire was realized using SoSci Survey [49] and made available to participants at www.soscisurvey.de. We were particularly interested in knowing whether the website covers the relevant topics for students, is understandable and attractive. The questionnaire consisted of 13 questions on different topics: First, we asked which chapters the participants had read (“chapters read”; one item), followed by questions concerning the “selection of topics” (two items; “yes”/“no” answer format). We then addressed the “attractiveness of the website” (five items). As we had given a lot of thought to different linguistic issues in advance, such as the length and complexity of the individual texts and the use of psychological terms, we also asked the students about the “comprehensibility of the website” (one item).

One challenge of the online portal is its preventive character. The aim is to motivate students to reflect on their behavior or even change their (inappropriate) behavior although they may not have encountered any difficulties during their studies so far. We were therefore interested in whether the website’s contents motivated the students to interact with the topics in greater depth, and asked about “impulses for action” (three items).

As the likelihood of recommending something to friends is an indicator of one’ s own satisfaction, we also asked about “recommendation to others” (one item). The topics “attractiveness of the website”, “comprehensibility of the website”, “impulses for action”, and “recommendation to others” were all rated on a 4-level scale from 1 “I do not agree” to 4 “I fully agree.”

There was no incentive to fill in the questionnaire.

## 3. Results (Status: 24.06.2020)

### 3.1. Website Usage

From October 2019 to June 2020, the website was visited 5739 times, 2357 of which were very short visits of 0–10 s. Excluding the ultra-short visits, 855 of the remaining were recurring visits with an average length of stay of 8 min and 18 s. Of the 2527 one-time visits, the average length of stay was 4 min and 35 s. There were, in total, 16,495 page views (see also Table 2) and 3748 downloads. Overall, 41% of visitors left the website after only one page.

The most frequently visited chapter is the chapter “well organized”, which is about time and stress management. Consistent with this result, the three most frequently downloaded information- and worksheets are from this chapter. The most frequently downloaded worksheet is a time management skill for creating a plan for the week (314 downloads), followed by an information sheet about typical “time killers” (e.g., using a smartphone, roommates causing disruptions while studying for an exam) and strategies to overcome them (254 downloads). The third most common download was a template for a monthly project plan (210 times), which is also a time management skill (all data collected via Matomo). 

### 3.2. Short Survey

**Figure 2:**
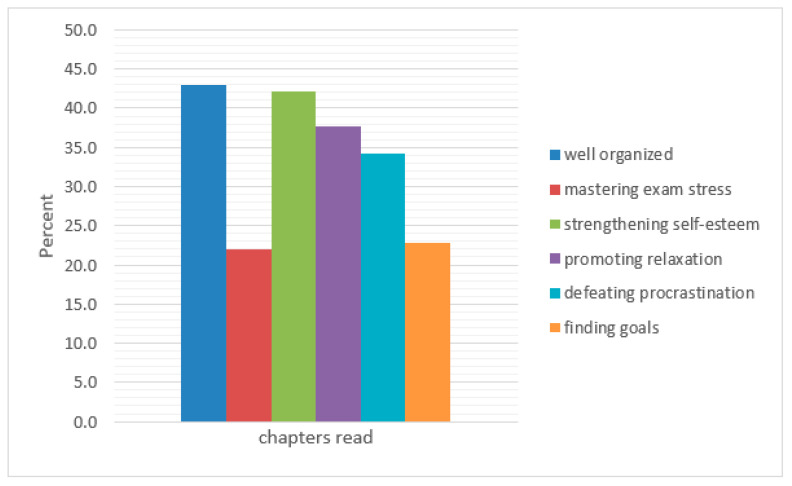
Chapters read (multiple answers possible; *N =* 114).

*N* = 372 website visitors clicked on the survey link; *N* = 114 questionnaires were completed fully. Most of the respondents looked at the chapters “well organized” (43%) and “strengthening self-esteem” (42.1%), see also Figure 2.

#### 3.2.1. Selection of Topics

**Figure 3:**
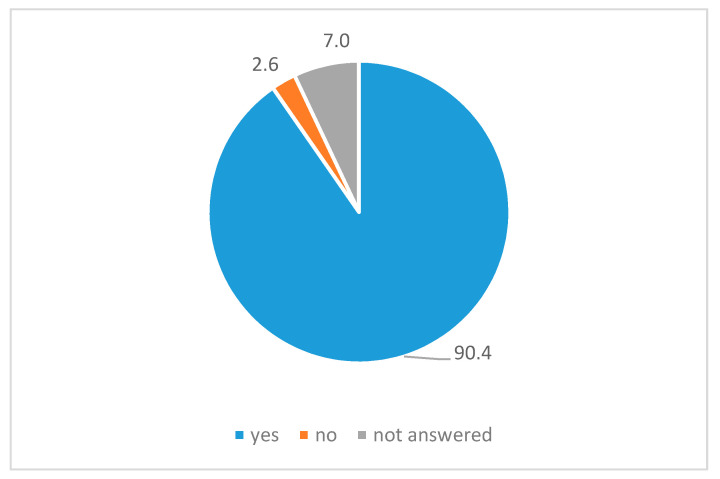
Response rate to the statement “The website addresses problems that relate to me”, in percent, *N =* 114.

The majority of respondents indicated that the website addresses problems that affect them (90.4%, Figure 3) and 93.9% felt that it dealt with issues that affect everyday life at university.

#### 3.2.2. Attractiveness of the Website

Altogether, 62.3% of the participants fully agreed with having had fun reading the website and more than half of the participants indicated that the layout of the website is visually appealing. The presentation of the texts on the website is considered attractive. Since the content is conveyed in different ways, there is something for every type of learner (texts to read, audio files and videos), which students evaluate as good (see Table 3). 

#### 3.2.3. Comprehensibility of the Website

**Figure 4:**
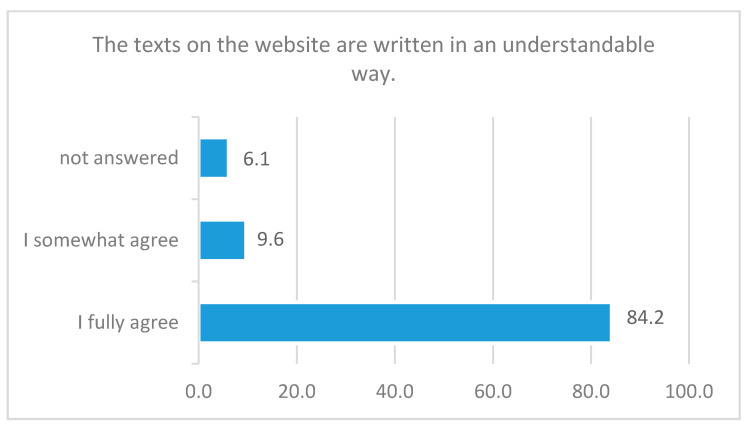
Response rate to the statement “The texts on the website are written in an understandable way”, in percent, *N =* 114.

The answers concerning the comprehensibility of the texts and information are mostly positive and show that the language of the texts is understandable and appropriate for this age group (Figure 4).

#### 3.2.4. Impulses for Action

Overall, 38.6% of the participants indicated that they wanted to implement some of the tips and 45.6% received new inspiration through the website. In addition, 59.6% would look at the website again if they needed some support at a later date (see Table 4).

#### 3.2.5. Recommendation to Others

**Figure 5:**
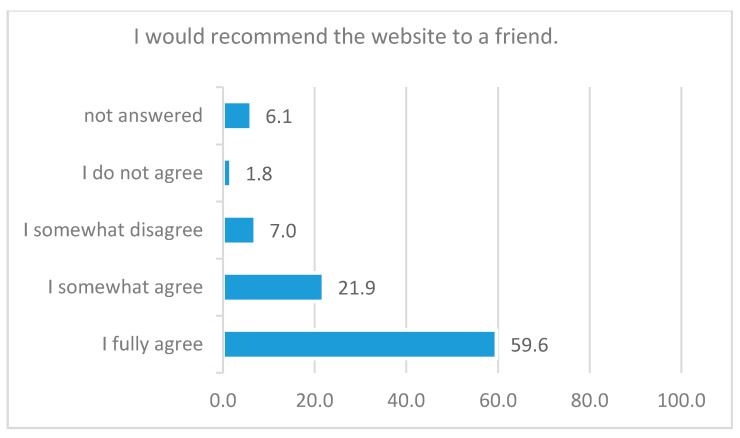
Response rate to the statement “I would recommend the website to a friend”, in percent, *N =* 114.

In total, 81.6% of the respondents would recommend me@JGU (see Figure 5).

## 4. Discussion

In this article, we presented the development, implementation and an initial usability evaluation on the use of me@JGU, a website with information on dealing with common stressors and mental health promotion for students. In summary, it can be said that we have succeeded in arousing curiosity and interest in the topic of mental health among students. The results of the survey show that the website covers topics that concern and interest the students (see Figure 3). This, in turn, indicates that it was helpful to carry out a needs analysis in advance. The answers also show that the layout of the website is visually appealing and the students rate the website as attractive (see Table 3), which encourages us in our decision to engage an agency for the realization so the website stands out from other teaching offers of the university. The students perceived the website as highly useful and highly user-friendly. These two factors contribute to user acceptance and help explain and predict user behavior of information technology [44].

The objective measures of engagement collected by the web usage data, showed that the website achieves high and continuous click rates, which we are very pleased about (Table 2). However, here too, as reported by other unaccompanied self-help offers, a relatively high drop-out rate of 41% is evident that we cannot explain (see Section 3.1). To our knowledge, there are no comparable naturalistic studies on usage and drop-out rates for unguided mental health promotion websites.

However, in an internet-based program to improve the mental health and well-being of young men, only approximately 10% of the website visitors registered to the program and only 11.7% completed the program [50]. Furthermore, Richards and Richardson [40] found a higher drop-out rate for unguided web-based interventions for depression (level of adherence 26%) compared to guided web-based interventions (level of adherence 72%). Spek and colleagues [51] examined 12 RCT studies on the treatment of depression and anxiety disorders in a meta-analytic study and found drop-out rates between 3% and 34%. Considering the results on drop-outs in Massive Open Online Courses (MOOCs), drop-out rates of up to 90% can be found [52]. MOOCs are courses that provide learning content—mostly of a psychoeducational character—online and can be taken without attendance restrictions, and are therefore comparable to the open, unaccompanied character of me@JGU. Perhaps the high drop-out rate of the me@JGU website could be partly explained by students who only briefly call up the page out of interest and read through it at a later time. Unfortunately, this is not interpretable by our click figures/data. Nevertheless, the students who stay on the website seem to read the content, download further information and come back again later (see also Section 3.1). The majority of the survey participants indicate that they are planning to implement the tips in everyday life (Table 4). This means that there are some students who are intensively engaged with the topics and benefit from the offer.

Unfortunately, we cannot draw any conclusions on the efficacy of our program yet. Various randomized-controlled trials have shown similar programs to be effective in reducing distress in college students [32,33,34,35,36,37]. Proof of the efficacy of our naturalistic and unguided setting remains to be shown. 

A further benefit is that through this website, offers of help, such as Mental Health Services for Students, are made known to students and any barriers towards help-seeking can be reduced.

### 4.1. Future Perspective

We plan to translate the website into English in order to also provide support for international students at JGU. In addition, a further evaluation of the usefulness and attractiveness of the website will be carried out. Another interesting point would be to examine more closely who benefits from and uses the information on the website and why. In the present study, we focused on early stage evaluations regarding usability and attractiveness. Subsequent testing should include issues like engagement, adherence and effectiveness. Pilot interviews with a small group of users could help make adjustments to the program before a time- and cost-intensive study is conducted.

The maintenance and further development of the project is also to be integrated into everyday counseling work of the Mental Health Services at JGU. Permanent technical support, e.g., for marketing purposes, and adaptation to current conditions (e.g., in April 2020, we created a subpage on the topic of Corona and mental health in studies) seems necessary and important to us. Currently, only JGU students have access to the website. Opening the offer for other students is under consideration.

### 4.2. Limitations

A limiting factor of the present study is that taking part in the survey was uncontrolled. The collected data therefore is selective, as the participants in an evaluation/survey are often those who are satisfied with a product. In addition, the online questionnaire was completed by only approximately 2% of the website users. This limits the generalizability of the findings. Furthermore, we cannot draw any conclusions concerning the efficacy of our program in promoting mental health.

## 5. Conclusions

Chronic stress in students can cause mental health problems and, therefore, have a negative impact on academic participation and outcomes.

The website me@JGU has been shown to cover topics that interest the students, is visually appealing, and achieves high and continuous click rates.

Initial evaluation results indicate that the website me@JGU provides impulses to engage with the website’s content.

Further studies addressing efficacy and adherence are needed.

## Figures and Tables

**Table 1:** Titles and contents of the eight chapters of the me@JGU website.

Chapter Title	Chapter Contents Based on Cognitive Behavioral Therapy	Examples of Downloads, Tips, Videos
1. Well organized	stress and time management	“Why we get stressed—and what effect it has on us.”; “Boosting your energy.”; “Your weekly schedule.”; “12-month project plan.”; “Time-eaters at university.”; “Be bold and set priorities.”
2. Mastering exam stress	techniques for test preparation, learning techniques, tips for presentations, lectures and oral exams, tips for academic writing	“Proper planning.”; “Learning techniques.”; “Mind-blanking—when your brain shuts down.”; “Rehearse an exam.”; “Being confident in public.”; “Writing exercises.”
3. Strengthening self-esteem	psychoeducation on self-esteem, strategies to improve self-esteem and self-care	“Self-esteem and self-evaluation.”; “Typical phrases that can influence your self-esteem.”; “My achievements, strengths, weaknesses.”; “Get to know your perfectionism.”; “Looking after yourself.”; “Self-esteem and body image.”; “A ‘positive events’ journal.”
4. Promoting relaxation	relaxation techniques, mindfulness exercises	“Exercise combats stress.”; “Get outdoors.”; “Progressive muscle relaxation.”; “Breath relaxation.”; “Mindfulness in daily university life.”
5. Managing negative feelings and thoughts	psychoeducation about the development of feelings, strategies for dealing with negative thoughts, strategies for regulating emotions	“Observe your thoughts.”; “Core beliefs influence your (automatic) thoughts.”; “Your core beliefs.”; “Typical thinking errors.”; “Modify your thoughts.”; “Reason is a tiger, emotion is a snail.”; “Be mindful of your emotions.”; “Controlling your emotions.”; “Emotional vulnerability.”
6. Your social network	strategies for making new contacts, maintaining relationships and dealing with conflicts	“Reviewing your social network.”; “Making new friends.”; “Maintaining your social network.”; “Expressing wishes.”; “Saying no.”; “Dealing with conflict.”; “Meeting with your supervisor/mentor.”
7. Defeating procrastination	psychoeducation on the vicious circle of procrastination, motivation strategies, dealing with obstructive thoughts	“The vicious circle of procrastination.”; “Pros and cons: Procrastination”; “Motivation strategies for routine use at university.”; “Using small time windows.”; “Procrastinating and postponing—there’s a big difference”
8. Finding goals	strategies to develop values and goals	“What are you passionate about?”; “A perfect day.”; “Your values.”; “Properly formulating your goals.”; “Dealing with setbacks.”

**Table 2:** (Selected) page views of the chapters of me@JGU website. ^1^

Chapter	Number of Page Views	Average Time per Page (min)	Minimum (min)	Maximum (min)
Well organized	1647	02:39	0 ^2^	55
Strengthening self-esteem	1056	02:05	0	56
Defeating procrastination	909	01:47	0	18
Promoting relaxation	837	01:21	0	24
Mastering exam stress	701	01:42	0	17
Finding goals	495	01:50	0	35

^1^ The table shows only the data for the content chapters of the website. ^2^ 0 min means that there were days during the evaluated time period, during which there were no visitors on the page.

**Table 3:** Response rates to the statements about the attractiveness of the website (in percent, *N =* 114).

Item	I Do Not Agree	I Somewhat Disagree	I Somewhat Agree	I Fully Agree	Not Answered
It was fun to look at the website.	-	5.3	22.8	62.3	6.1
I like the website./The website is attractive.	0.9	1.8	23.7	60.5	6.1
The texts on the website are presented in an interesting way.	-	4.4	20.2	68.4	7.0
I like that audio files are offered.	3.5	7.0	21.1	51.8	9.6
I like the podcast.	0.9	9.6	31.6	28.1	22.8

**Table 4:** Response rates to the statements concerning impulses for action (in percent, *N =* 114).

Item	I Do Not Agree	I Somewhat Disagree	I Somewhat Agree	I Fully Agree	Not Answered
I think that I will put some tips into practice.	0.9	6.1	44.7	38.6	6.1
I have received new ideas/information.	4.4	12.3	28.1	45.6	6.1
I would return to the website if I needed help.	3.5	9.6	17.5	59.6	6.1

**Figure A1:**
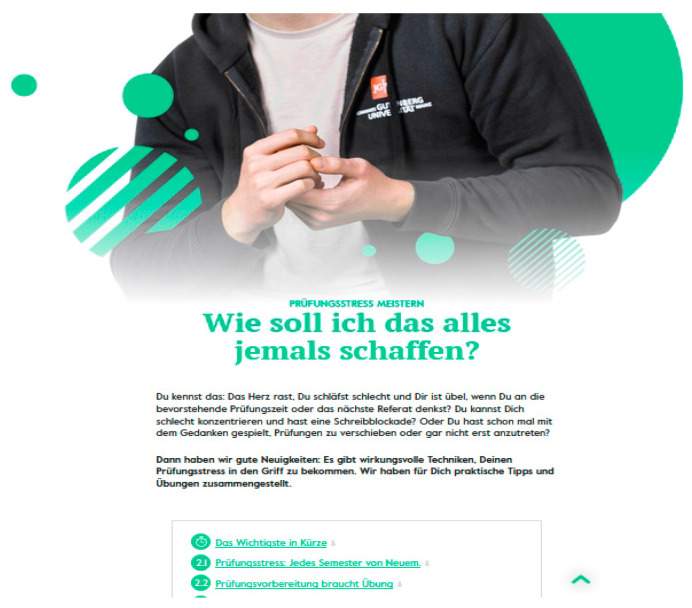
Chapter 2 “Mastering exam stress”.

**Figure A2:**
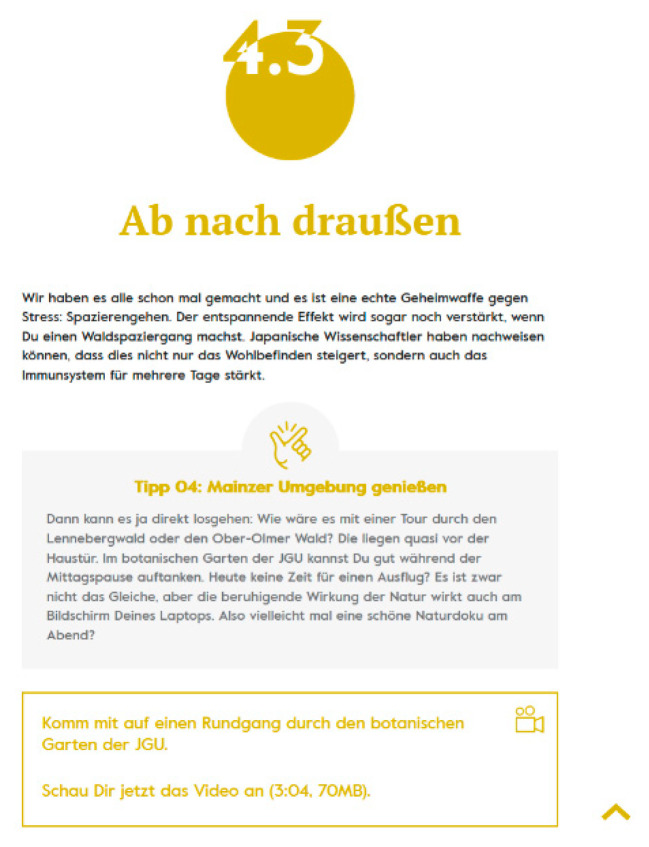
Chapter 4“Promoting relaxation”.

**Figure A3:**
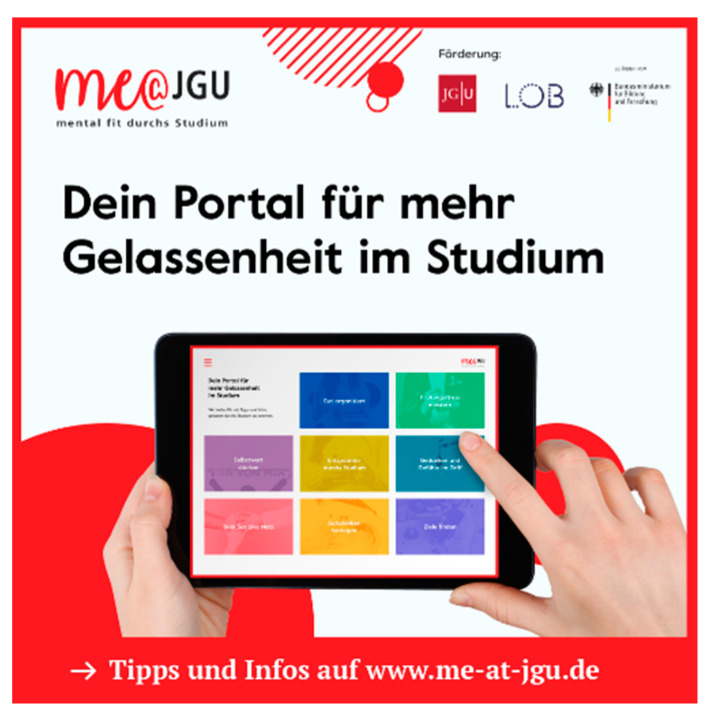
An advertising banner for Social Media.

## Data Availability

The data presented in this study are available on request from the corresponding author.

## Appendix A

Two Screenshots of the website (me@JGU)
